# Refining the definition of HER2‐low class in invasive breast cancer

**DOI:** 10.1111/his.14780

**Published:** 2022-09-12

**Authors:** Nehal M Atallah, Michael S Toss, Andrew R Green, Nigel P Mongan, Graham Ball, Emad A Rakha

**Affiliations:** ^1^ Department of Histopathology School of Medicine, the University of Nottingham and Nottingham University, Hospitals NHS Trust Nottingham UK; ^2^ Department of Pathology Faculty of Medicine, Menoufia University Menoufia Egypt; ^3^ Division of Cancer and Stem Cells Biodiscovery Institute, School of Medicine, University of Nottingham Nottingham UK; ^4^ Histopathology Department Royal Hallamshire Hospital, Sheffield Teaching Hospitals NHS Foundation Trust Sheffield UK; ^5^ School of Veterinary Medicine and Sciences University of Nottingham Sutton Bonington UK; ^6^ Division of Life Sciences Nottingham Trent University Nottingham UK

**Keywords:** ANN, breast cancer, HER2 low, *HER2* mRNA, refining

## Abstract

**Background:**

Emerging evidence indicates that breast cancer (BC) patients whose tumours express HER2 protein without *HER2* gene amplification (HER2‐low), can benefit from antibody–drug conjugates (ADC). However, the current definition of HER2‐low BC remains incomplete with low rates of concordance. This study aims to refine HER2‐low definition with emphasis on distinguishing HER2 score 0 from score 1+ to identify patients who are eligible for ADC.

**Methods:**

A BC cohort (*n* = 363) with HER2 IHC scores 0, 1+ and 2+ (without *HER2* gene amplification) and available *HER2* mRNA was included. HER2 staining intensity, pattern and subcellular localisation were reassessed. Artificial neural network analysis was applied to cluster the cohort and to distinguish HER2 score 0 from 1+. Reproducibility and reliability of the refined criteria were tested.

**Results:**

HER2 IHC score 1+ was refined as membranous staining in invasive cells as either: (1) faint intensity in ≥ 20% of cells regardless the circumferential completeness, (2) weak complete staining in ≤ 10%, (3) weak incomplete staining in > 10% and (4) moderate incomplete staining in ≤ 10%. Based on this, 63% of the HER2‐negative cases were reclassified as positive (HER2‐low). The refined score showed perfect observer agreement compared to the moderate agreement in the original clinical scores. Similar results were generated when the refined score was applied on the independent BC cohorts. A proposal to refine the definition of other HER2 classes is presented.

**Conclusion:**

This study refined the definition of HER2‐low BC based on correlation with *HER2* mRNA and distinguished between HER2 IHC score 1+ and score 0 tumours.

## Graphical abstract

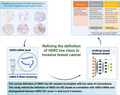

## Introduction

*Human epidermal growth factor receptor 2 (HER2)* gene is amplified in approximately 15% of invasive breast cancer (BC) leading to HER2 protein overexpression.^1^, ^2^, ^3^, ^4^ HER2 testing in routine practice is performed using immunohistochemistry (IHC) to assess the level of protein expression, which is reported using a range of 0 to 3+ score.^5^, ^6^ HER2‐positive BC is defined as IHC score 3+ or score 2+ with evidence of *HER2* gene amplification using the *in‐situ* hybridisation (ISH) technique. HER2‐positive BC patients are eligible for therapies that target the HER2 pathways.^7^, ^8^, ^9^ BC with HER2 IHC score 2+ that lacks evidence of *HER*2 gene amplification is currently classified as HER2‐negative similar to cases showing IHC score of 0 or 1+^5^, ^6^ and do not benefit from anti‐HER2 therapy. However, recent data have demonstrated that some of the HER2 directed antibody–drug conjugates (ADC) such as trastuzumab–emtansine (T‐DM1) and trastuzumab–deruxtecan (T‐DXD) can improve the outcome of patients with BC that express HER2 protein without evidence of *HER2* gene amplification.^10^ These cases included BC with HER2 IHC score 1+ or score 2+ without *HER*2 gene amplification, which are defined as the HER2‐low class.^11^, ^12^, ^13^, ^14^

ADCs are molecules consisting of a recombinant monoclonal antibody covalently bound to a cytotoxic drug via a linker. After antibody binding to the specific antigen on the targeted cell surface, the cytotoxic drug becomes internalised and is released intracellularly, where it can exert its effect. ADC effect relies upon the presence an extracellular protein receptor which acts as a carrier for the cytotoxic agent to achieve targeted effect with no or minimal levels of cytotoxicity to the normal cells, rather than on the oncogenic effect of the protein. Patients’ recruitment to the ongoing HER2‐low‐positive clinical trials which are testing the effect of ADCs in BC are based on the existing definition of HER2 categories, as described in the American Society of Clinical Oncology and College of American Pathologists (ASCO/CAP).^6^

Although the ASCO/CAP guideline recommendations provided comprehensive definition of the HER2 staining pattern and the categorisation of cases into four IHC scores (0–3+), the distinction between IHC score 0 and 1+ is not sufficiently detailed and lacks relevant evidence, and some scenarios of HER2 expression patterns are missing.^5^, ^6^ This could participate in the high discordance rates in HER2 status assessments reported in some studies.^15^, ^16^, ^17^

Although clinical response can provide the best tool to define the lower limit of the HER2‐low class, the number of recruited patients in such randomised clinical trials, particularly those close to the threshold of positivity, is typically too limited to develop a robust definition. In this study, we have used a large cohort of BC that express low levels of HER2 protein without evidence of *HER2* gene amplification and applied an artificial neural network (ANN) model to refine the definition of HER2‐low class of BC with an emphasis on distinguishing HER2 score 1+ and 0 categories. We have used the *HER2* mRNA levels as a ground truth to reflect the level of *HER2* gene expression. ANNs can learn and model non‐linear and complex relationships.^18^, ^19^, ^20^, ^21^, ^22^ We have also tried to refine the existing definitions of HER2 IHC categories by completing the missing scenarios utilising the existing data and our experience.

## Materials and methods

This study was conducted on a primary invasive BC cohort (*n* = 363) from patients presenting at Nottingham University Hospitals NHS Trust with a HER2 IHC score 0, 1+ and 2+ without gene amplification. Transcriptomic data on *HER2* mRNA expression were available for this cohort within the recorded Oncotype DX report,^23^ which was carried out as part of the patients’ clinical care for management. Briefly, mRNA levels were obtained from tumour samples extracted from formalin‐fixed paraffin‐embedded tissue using high‐throughput, real‐time, reverse transcription–polymerase chain reaction. Normalised expression measurements were calculated as the mean cycle threshold (CT) for the five reference genes minus the mean CT of triplicate measurements for each individual gene*. HER2* mRNA level ranged between 5.0 and 10.8, units with a mean of 9 units.

The clinicopathological data, including age at diagnosis, tumour size, histological grade, histological tumour type, axillary lymph node status, lymphovascular invasion (LVI) and Nottingham prognostic index (NPI), were available (Supporting information, Table S1). The patients’ mean age at diagnosis was 59 years, while the mean invasive tumour size was 2.2 cm (range = 0.1–11.5 cm). All cases were oestrogen receptor (ER)‐positive and HER2‐negative. ER and progesterone receptor (PR)‐positivity were assessed according to ASCO/CAP guidelines if ≥ 1% of the invasive tumour cell nuclei were immunoreactive.^24^ HER2 staining was completed on the Ventana Benchmark ULTRA immunohistochemistry automated staining system using the Ventana PATHWAY anti‐HER‐2/*neu*, rabbit monoclonal ready‐to‐use primary antibody in combination with Ventana detection kits. No antigen retrieval was required, according to the protocol.

Appropriate positive and negative controls were included for each staining run as per the published guidelines.^5^, ^6^ Protein expression assessment was carried out in routine clinical practice using light microscopy on the diagnostic core needle biopsies. The reported HER2 scoring categories in the clinical setting were retrieved from the patient records.

### Detailed reassessment of HER2 IHC protein expression

HER2 expression within the invasive tumour cells only of each case was reassessed and presented in detail. This included: (1) cellular localisation of protein expression (membranous, cytoplasmic or both) and (2) intensity of staining divided into five grades (negative, faint, weak, moderate and strong). In addition to the comparison with the positive and negative controls, the magnification rule was used to guarantee high interobserver agreement. Strong HER2 staining was assessed as those cases displaying unequivocal membranous staining are seen easily at low‐power magnification (2× or 4×), while unequivocal membranous staining (moderate to weak) was only assigned at medium magnification (10× to 20×, respectively). Faint staining can only be appreciated at 40× magnification, whereas weak staining can be appreciated at 20× magnification.^25^ Cases were assessed using the NIKON NI‐U Microscope, Nikon UK, Surbiton, UK. Different intensities within the same tumour were assessed to reflect the heterogeneity, (3) the percentage of each intensity, (4) distribution/completeness of membranous staining as either complete circumferential membranous or incomplete lateral or basolateral staining and (5) histo score (H‐score) was calculated as follows: % of weak intensity × 1+% of moderate intensity × 2+% of strong intensity × (3). In addition, the % of faint intensity was assessed and multiplied by 0.5 to produce a total score of 350. Each incomplete membranous staining is multiplied by 0.5, while complete membranous staining is multiplied by 1.

### HER2 staining on full‐face sections of resection specimen

HER2 IHC staining and scoring were performed on core biopsies while *HER2* mRNA level was assessed on resection specimens. Thus, for the cases that showed a discrepancy between HER2 IHC score and mRNA level *(n* = 30), i.e. high mRNA level and HER2 score 0 or HER2 score 2+ with low mRNA level, repeating HER2 IHC staining on the full‐face sections using the same tissue block tested for Oncotype DX was performed. Whenever possible, the same tissue block that was used to run the Oncotype DX test was stained with HER2. The staining protocol was similar to the core biopsy staining as described above.

### Defining cut‐off for HER2 score 1+ versus score 0

**Figure 1:**
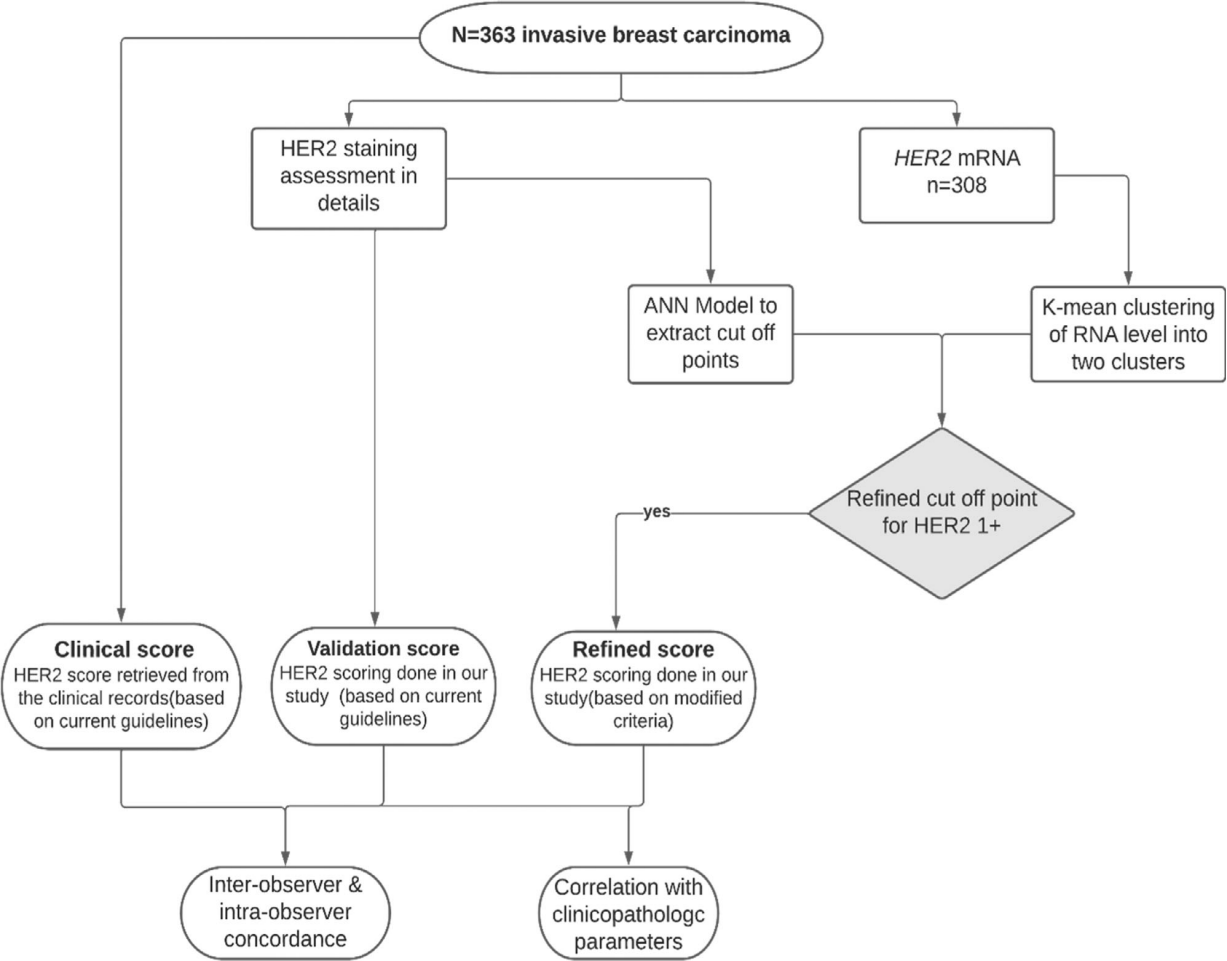
Flowchart summarising cohort selection and different steps carried out.

Two steps were followed to define HER2 score 1+ (Figure 1).

#### Step 1: K‐means clustering

The K‐means technique aims to partition the data into K‐groups such that the sum of squares from points to the assigned cluster centres is minimised. *HER2* mRNA values were classified, using K‐means, into two clusters based on their similarity of expression across multiple HER2 scoring parameters. Those cases which had a score of 1+ or 0 were clustered into two groups based on *HER2* mRNA level and the detailed IHC scoring performed. Cluster 1 was defined as HER2‐negative (0) while cluster 2 represented HER2‐positive (1+). HER2 2+ were excluded from the clustering to avoid data bias.

#### Step 2: Artificial neural network model (ANN)

The ANN model (NeuroSolution version 7.0; NeuroDimension, Gainesville, FL, USA), with a range of hidden nodes in three layers, with a Levenburg Marquardt algorithm and a TanH activation function), was used to set the cut‐off point for defining HER2 1+ based on the K‐means clusters defined in step 1. A Monte Carlo cross‐validation approach was used to train a population of models, and early stopping was undertaken using a randomly extracted unseen cross‐validation set with subsequent validation on a test set (*n* = 38), which was kept completely blind to training process. Weight regularisation was conducted during training.

The model was trained with the detailed HER2 scoring parameters, including various intensities (faint, weak, moderate) and distribution of each intensity, if present, either complete or incomplete, in addition to the total percentage of positive cells and cytoplasmic staining as input and *HER2* mRNA‐based clusters as an output variable. The ANN model determined which of the input parameters predicted HER2 score 1+ with a high level of accuracy. Sensitivity and specificity with the produced response curves was applied to set the cut‐off for the most participating parameter.

Predictions of trained models were examined to decide predicted probability of K‐means cluster membership. These were examined to determine a probabilistic cut‐point for HER2 score 1+. Model performance was further assessed by finding the area under the curve (AUC) of a constructed receiver operator characteristic (ROC) curve. AUCs of 1 were seen across the three cross‐validation cohorts as well as 100% classification rates.

After setting the cut‐points, a new refined score for HER2 was developed and applied. To detect the accuracy of our refined score against the clinical score, we used the same neural network to build a discriminating model of both HER2 scores using the clinicopathological parameters as input units and the HER2 score as an output. The differentiating performance of the ANN models was evaluated with AUC as well as the true‐ and false‐positivity rates.

To test the reliability of using *HER2* mRNA as a dichotomising variable, we assessed the correlation between *HER2* mRNA, protein level and gene amplification levels in a large independent cohort of primary BCs obtained from two publicly available data sets: the Cancer Genome Atlas (TCGA) (*n* = 614) and the Molecular Taxonomy of Breast Cancer International Consortium (METABRIC) (*n* = 288).

### Reliability and reproducibility of the refined HER2 IHC score

The efficiency and reproducibility of the refined HER2 scoring method against the current guidelines were tested. HER2 was scored twice according to the existing ASCO/CAP; once by the clinical team at time of diagnosis, and the second score was carried out by experienced pathologists (N.A. and M.T.) who have more than 5 years’ experience in histopathology, supervised by an experienced breast pathology consultant (E.R.) with more than 20 years’ experience in the field of breast pathology. The agreement between two scores was assessed. Moreover, the interobserver agreement of the refined score was assessed between both observers and the intra‐observer agreement was examined through rescoring the cases after a 3 months’ washout period.

### Correlation between refined HER2 score with the clinicopathological variables

The correlation between the clinicopathological parameters, including *HER2* mRNA level, HER2 scores including the refined and the original clinical scores, was carried out. In addition, *HER2* mRNA K‐means clusters were correlated with the other clinicopathological parameters.

### Statistical analysis

SPSS version 24 was used to carry out the statistical analysis. Correlations were analysed using χ^2^, Fisher's exact, Kruskal–Wallis and Wilcoxon rank sum tests with continuity correction, where appropriate. The concordance analysis was performed using Cohen's Kappa test. All differences were considered significant at *P* < 0.05.

## Ethics approval and consent to participate

Ethical approval was obtained for this study and approved by REC (ref. no. 19/SC/0363) under the title ‘PathLAKE’. All cases included in the study were fully anonymised.

## Results

### Patterns of HER2 protein expression

Of the cases in the study cohort, 81% showed a degree of HER2 expression regardless of the pattern and/or the percentage of positive cells. It was observed that each case had a mixture of expression patterns, intensities and cellular localisation. The most frequent pattern observed was incomplete faint staining, which presented in 78% of the cohort, followed by complete faint expression which presented in 58% of cases with or without other patterns of expression. Moderate incomplete staining had the lowest proportion among all patterns of expression (4%). Detailed description of HER2 expression in terms of staining intensities, patterns and percentages are summarised in (Supporting information, Table S2 and Figure S1).

### Correlation between HER2 IHC score and mRNA level

Discrepancy between HER2 IHC protein expression score on core biopsy and mRNA level was seen in 30 of 363 (8%) of cases. IHC score 2+ with low mRNA level, as defined based on K‐mean clustering analysis, was presented in (two of 30) cases, while the remaining 28 cases had IHC score 0 with high mRNA level. Upon staining those 30 cases on full‐face tissue sections, all cases that were scored 0 on core biopsy were completely negative (score 0) in the invasive tumour cells with weak to moderate staining within the *in‐situ* component, while the two cases that were score 2+ in core biopsy emerged as completely negative (score 0) on full‐face sections.

### Clustering of *HER2* mRNA

A total of 308 cases with complete data on different HER2 expression patterns and *HER2* mRNA level were available for K‐mean clustering analysis. The data set was divided into two clusters; cluster 1 (*n* = 109) and cluster 2 (*n* = 199), based on mRNA level at cut‐off 8.7 units. Based on the new cut‐off, the mean values ± standard deviation (SD) of *HER2* mRNA in HER2 in HER2 IHC were 0, 1+ and 2+ was 8.66 ± 0.69, 9.1 ± 0.66 and 9.4 ± 0.78 (Supporting information, Figure S2).

### ANN model sensitivity

**Figure 2:**
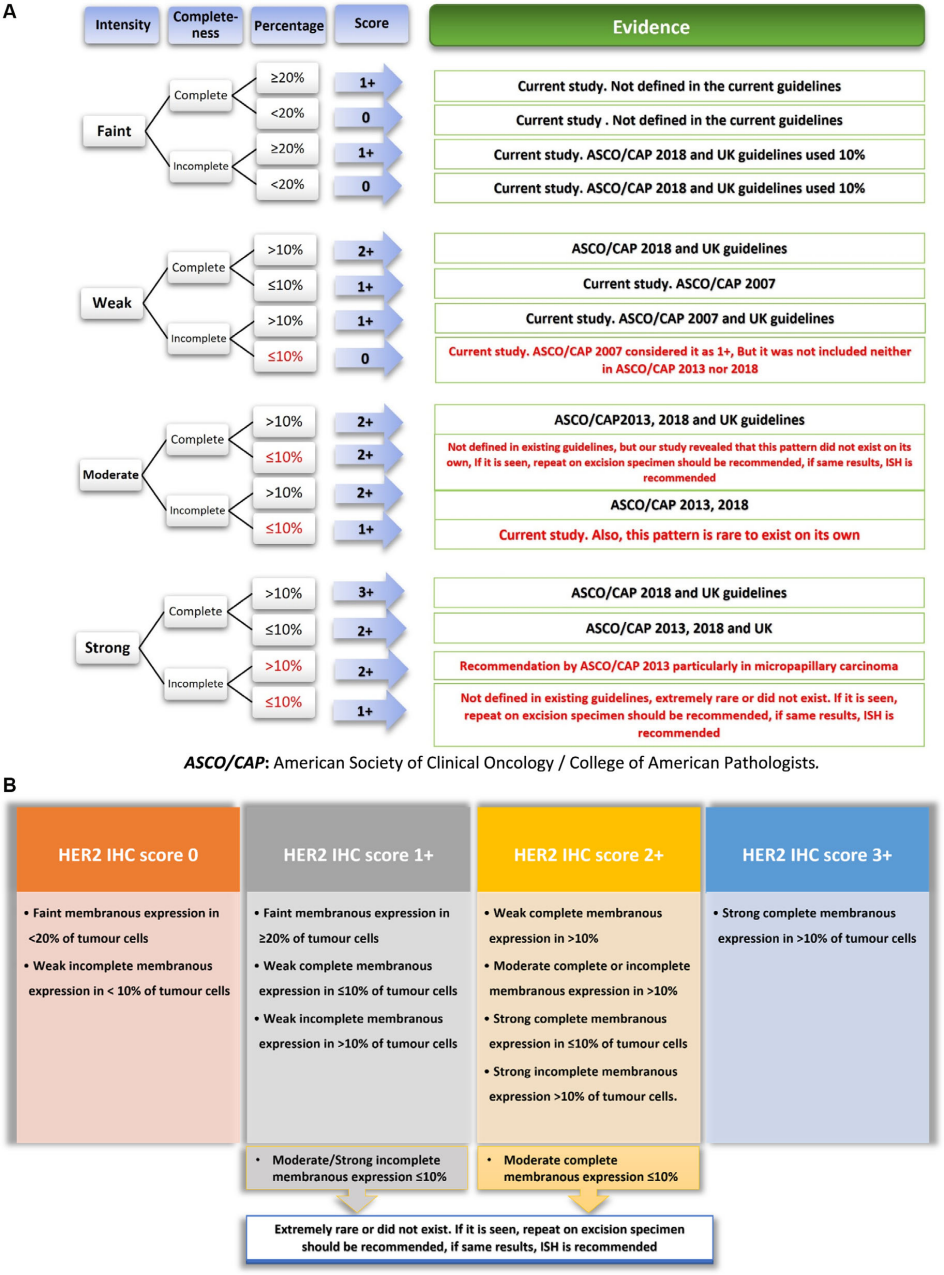
**A,** Schematic illustration of different scenarios of human epidermal growth factor receptor 2 (HER2) expression in breast cancer and the corresponding score from different existing guidelines. **B,** Recommended HER2 scoring algorithm based on immunohistochemistry (IHC)‐stained slides.

The parameters that predicted HER2 cluster 2 (equivalent to score 1+) were faint complete staining at ≥ 20% of invasive tumour cells and/or faint incomplete staining in ≥ 20%, weak complete staining in ≤ 10%, weak incomplete staining in > 10% and moderate incomplete membranous staining in ≤ 10% of invasive tumour cells. Cytoplasmic staining and total percentage of positive cells did not define the cluster (Table 1, Supporting information, Figure S3). Figure 2A,B shows a schematic illustration of different scenarios of HER2 expression in BC and the corresponding score based on the refined criteria compared to the existing guidelines.^5^, ^6^, ^26^, ^27^

**Table 1:** Comparison between HER2 score 1+ definition based on the existing guidelines against the refined criteria

Refined definition	Existing ASCO/CAP and UK guidelines
*Faint complete and/or faint incomplete in ≥ 20%	Incomplete membrane staining that is faint/barely perceptible and in > 10% of tumour cells
Weak complete ≤ 10%	
Weak incomplete > 10%	
Moderate incomplete < 10%	

ASCO/CAP, American Society of Clinical Oncology /College of American Pathologists; HER2, human epidermal growth factor receptor 2.

* Total faint staining in tumour cells ≥ 20%.

Based on the new defined cut‐points for low HER2 IHC scoring, 136 of 363 (37%) cases were scored 0, 140 of 363 cases (39%) with score 1+ and 87 of 363 cases (24%) were scored 2+ compared to 126 of 363 (35%), 156 of 363 (43%) and 81 of 87 (22%) for the original scores 0, 1+ and 2+, respectively (Supporting information, Figure S2).

Faint staining intensity was the most predominant pattern in HER2 score 1+ (41 of 140), followed by weak incomplete staining in > 10% of invasive tumour cells and then weak complete staining of less than or equal to 10% of cells. Exclusive moderate expression, either complete or incomplete, < 10% was not found in HER2 1+ as a unique pattern, but was expressed in combination with other patterns (Figure 31,2).

**Figure 3:**
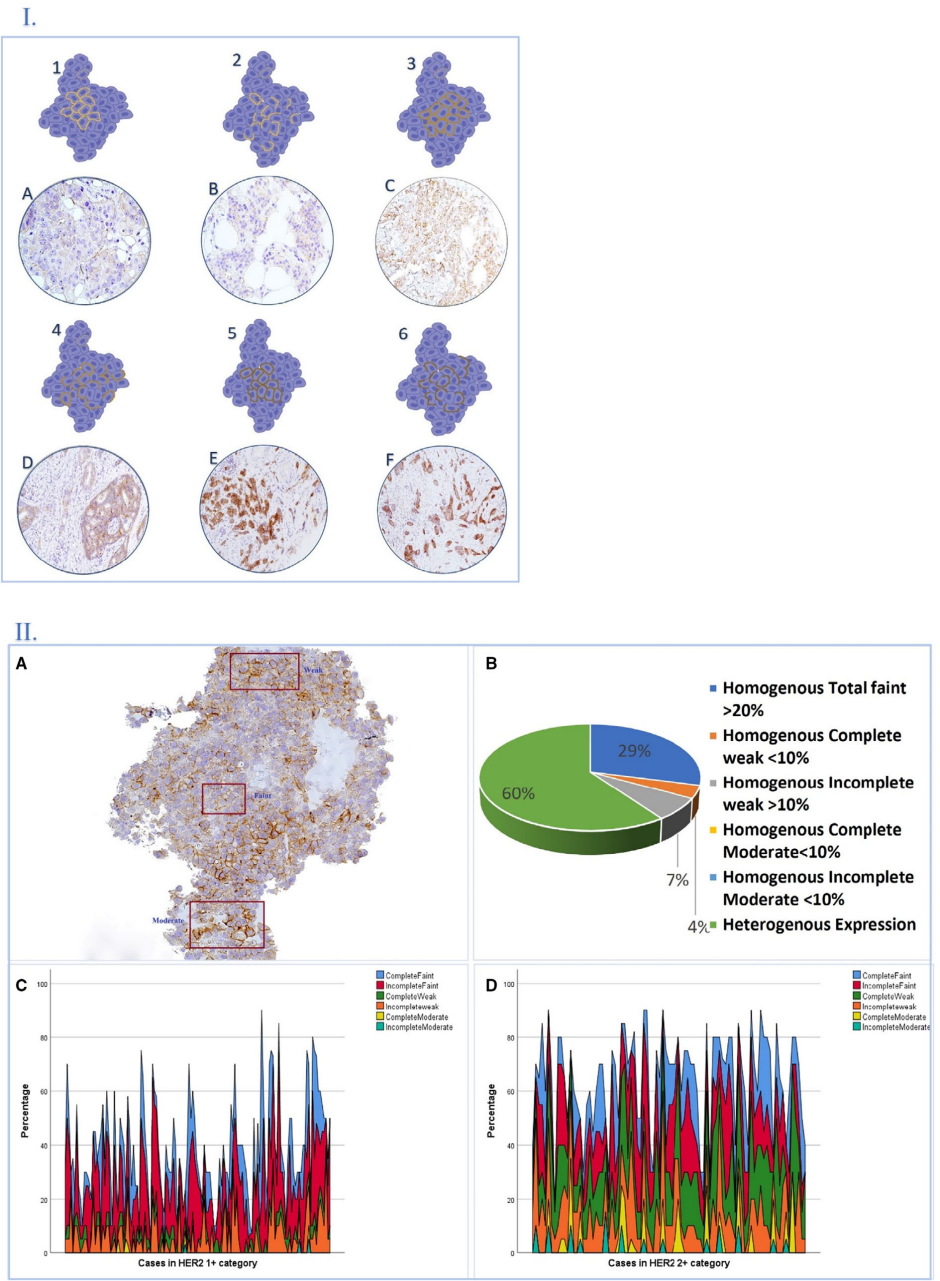
**1,** An illustrated diagram and photomicrographs showing different human epidermal growth factor receptor 2 (HER2) membranous staining patterns and intensities. Faint complete **(1A)**; faint incomplete **(2B)**; weak complete **(3C)**; weak incomplete **(4D)**; moderate complete **(5E)**, moderate incomplete **(6F)**. **2,** Graphical description highlighting degree of intratumoral heterogeneity HER2) low category. **2A,** HER2 immunohistochemistry (IHC)‐stained slide showing different staining intensities within the same tumour; **2B,** pie chart showing that 60% of HER2 1+ cases were scored based on heterogenous mixed expression patterns, while only 40% were scored based on single homogenous staining. Moderate staining was present in addition to other staining patterns and not in the HER2 1+ category alone. Sparkline graphs show multiple combinations of heterogenous patterns in example of HER2 score 1+ (**2C**), and another example of HER2 score 2+ (**2D**).

The AUC for the refined score was 0.92 with a true‐positive rate of 92% and false‐positive rate of 13%. The AUC for the original clinical score was 0.71, with 69 and 34% as true‐positive rates and false‐positive rates, respectively (Supporting information, Figure S4).

### Reproducibility of the refined HER2 IHC score

The degree of concordance between the score given on the original clinical settings and the redefined score upon applying the existing HER2 scoring criteria was substantial (kappa = 0.6). Exact score agreement was 79%, while the number of discordant cases was 75 of 363 cases (20%); 47 were between IHC score 1+ versus 0 and the remaining 28 discordant cases were between 1+ versus 2+. None of the cases showed score 2+ versus 0 discrepancy. Regarding the refined score, the intra‐observer concordance showed perfect agreement (kappa = 0.8) with 87% exact score agreement. Furthermore, the interobserver agreement was perfect (kappa = 0.9), with 89% exact score agreement. Overall, there were 36 of 363 cases (10%) with discordance. Table 2 details the agreement levels. There was a strong association between H score and HER2 IHC scores (*P* < 0.001).

**Table 2:** Concordance rates of the refined HER2 score

Interobserver concordance of the refined HER2 score between the two observers					
Second observer score	First observer score				Kappa value
	0	1	2	Total	
0	128 (93.4%)	9 (6.6)	0	137	0.85
1	8 (5.7)	122 (87.1%)	10 (7%)	140	
2	0	9 (10.5%)	77 (89.5%)	86	
Total	136	140	87	363	
Intra‐observer agreement of HER2 score using the refined criteria					
Refined HER2 score (second)*	Refined HER2 score (first)				0.83
	0	1	2	Total	
0	124 (92%)	11 (8%)	0	135	
1	12 (8.3%)	119 (82.6%)	13 (9%)	144	
2	0	10 (12%)	74 (88%)	84	
Total	136	140	87	363	

* Carried out by the first observer after 3 months washout period from the 1st score. HER2, human epidermal growth factor receptor 2.

### The association between various clinicopathological parameters and the refined HER2 score in comparison with the original score

Both HER2 scores showed a statistically significant correlation with lymph node status and mRNA level (Table 3). The refined score, but not the original score, showed a statistically significant correlation with Oncotype DX recurrence score (*P* = 0.02), where score 0 was associated with higher‐risk Oncotype DX groups. Moreover, there was a statistically significant difference between HER2 IHC 1+ and 2+ using the refined score regarding lymph node metastasis, where IHC 1+ was associated with lymph node metastasis. When compared to IHC score 0 cases, there was statistically significant correlation between HER2‐low cluster and low tumour grade (*P* < 0.001), lower pleomorphism score (*P* = 0.001) and low mitotic count (*P* < 0.001), less DCIS within tumour (*P* < 0.001) and more lymph node metastasis (*P* = 0.03) (Supporting information, Table S3).

**Table 3:** Associations between clinicopathological parameters and HER2 scores (using the refined and the original clinical score)

Parameter	Refined HER2 score						Clinical HER2 score					
	HER2 IHC 0 (*n*, %)	HER2 IHC 1+ (*n*, %)	HER2 IHC 2+ (*n*, %)	*P‐*value^a^	*P‐*value^b^	*P‐*value^c^	HER2 IHC 0 (*n*, %)	HER2 IHC 1+ (*n*, %)	HER2 IHC 2+ (*n*, %)	*P‐*value^a^	*P*‐value^b^	*P‐*value^c^
Age at diagnosis (years)												
< 50	36 (44)	24 (29)	22 (27)	χ^2^ = 3.52	χ^2^ = 2.20	χ^2^ = 1.87	26 (32)	43 (53)	12 (15)	χ^2^ = 1.81	χ^2^ = 4.50	χ^2^ = 0.36
≥ 50	100 (36)	116 (41)	65 (23)	0.079	0.140	0.170	100 (36)	113 (40)	67 (24)	0.21	**0.035**	0.548
Menopausal status												
Pre	35 (34)	44 (44)	22 (22)	χ^2^ = 1.09	χ^2^ = 0.98	χ^2^ = 0.47	37 (37)	40 (40)	24 (23)	χ^2^ = 0.49	χ^2^ = 0.59	χ^2^ = 0.19
Post	101 (38)	96 (37)	65 (25)	0.290	0.320	0.490	89 (34)	116 (45)	55 (21)	0.500	0.440	0.710
Tumour size (cm)												
< 2	57 (34)	71 (43)	39 (23)	χ^2^ = 2.14	χ^2^ = 0.74	χ^2^ = 1.47	62 (37)	64 (38)	41 (25)	χ^2^ = 1.88	χ^2^ = 2.50	χ^2^ = 0.67
≥ 2	79 (40)	69 (35)	48 (25)	0.149	0.420	0.230	64 (33)	92 (47)	38 (20)	0.186	0.128	0.440
Associated DCIS												
No	33 (30)	49 (45)	28 (25)	χ^2^ = 3.87	χ^2^ = 0.19	χ^2^ = 3.80	34 (31)	52 (48)	23 (21)	χ^2^ = 1.33	χ^2^ = 0.43	χ^2^ = 0.95
Yes	103 (41)	91 (36)	59 (23)	0.051	0.660	0.053	92 (37)	104 (41)	56 (22)	0.300	0.510	0.340
Histological tumour type												
NST	87 (37)	86 (37)	60 (26)	χ^2^ = 0.191	χ^2^ = 1.33	χ^2^ = 0.04	82 (35)	95 (41)	56 (24)	χ^2^ = 0.52	χ^2^ = 2.28	χ^2^ = 0.03
Other types	49 (38)	54 (42)	27 (21)	0.709	0.250	0.940	44 (34)	61 (48)	23 (18)	0.536	0.130	0.900
Histological tumour types												
NST	87 (37)	86 (27)	60 (26)				82 (35)	95 (41)	56 (24)			
Lobular	20 (36)	23 (42)	12 (22)	χ^2^ = 0.04	χ^2^ = 2.82	χ^2^ = 1.04	19 (35)	29 (53)	7 (13)			
Tabular	3 (27)	5 (46)	3 (27)	0.851	0.590	0.900	3 (27)	4 (36)	4 (36)	χ^2^ = 0.20	χ^2^ = 0.92	χ^2^ = 0.07
Special type	23 (40)	23 (40)	12 (20)				20 (36)	24 (43)	12 (21)	0.660	0.340	0.930
Metaplastic carcinoma	3 (50)	3 (50)	0 (0)				2 (33)	4 (67)	0 (0)			
Histological tumour grade												
1	6 (26)	13 (57)	4 (17)				8 (35)	9 (40)	6 (26)			
2	79 (39)	76 (37)	48 (24)	χ^2^ = 2.58	χ^2^ = 1.06	χ^2^ = 1.46	70 (35)	95 (47)	36 (18)	χ^2^ = 0.82	χ^2^ = 5.01	χ^2^ = 0.02
3	51 (37)	51 (37)	35 (26)	0.275	0.302	0.480	48 (35)	52 (38)	37 (27)	0.662	0.080	0.900
Tubule formation												
1	4 (33)	6 (50)	2 (17)	χ^2^ = 1.16	χ^2^ = 0.04	χ^2^ = 1.48	4 (33)	5 (42)	3 (25)	χ^2^ = 3.00	χ^2^ = 1.17	χ^2^ = 1.40
2	21 (31)	27 (40)	19 (28)	0.286	0.841	0.480	18 (27)	35 (53)	13 (20)	0.173	0.560	0.240
3	111 (39)	107 (38)	66 (23)				104 (37)	116 (41)	63 (22)			
Pleomorphism*												
2	58 (42)	50 (37)	29 (21)	χ^2^ = 1.31	χ^2^ = 0.13	χ^2^ = 2.20	54 (40)	55 (40)	28 (20)	χ^2^ = 1.70	χ^2^ = 0.01	χ^2^ = 2.00
3	78 (35)	90 (40)	58 (26)	0.268	0.775	0.140	72 (32)	101 (45)	51 (23)	0.219	0.980	0.170
Mitosis												
1	77 (37)	82 (40)	47 (23)	χ^2^ = 1.62	χ^2^ = 3.68	χ^2^ = 0.23	73 (36)	93 (46)	38 (19)	χ^2^ = 0.08	χ^2^ = 2.80	χ^2^ = 0.16
2	32 (36)	38 (43)	19 (21)	0.414	0.159	0.890	30 (34)	36 (40)	23 (26)	0.960	0.243	0.920
3	27 (40)	20 (29)	21 (31)				23 (34)	27 (40)	18 (27)			
LVI												
No	104 (36)	115 (40)	69 (24)	χ^2^ = 1.31	χ^2^ = 0.28	χ^2^ = 1.09	101 (35)	121 (42)	65 (23)	χ^2^ = 0.28	χ^2^ = 0.71	χ^2^ = 0.05
Yes	32 (43)	25 (33)	18 (24)	0.298	0.600	0.300	25 (34)	35 (47)	14 (18)	0.662	0.400	0.890
Lymph node status												
Negative	97 (41)	77 (33)	63 (27)	χ^2^ = 7.54	χ^2^ = 6.57	χ^2^ = 3.30	93 (40)	95 (40)	48 (20)	χ^2^ = 5.23	χ^2^ = 0.09	χ^2^ = 6.06
Positive	39 (31)	62 (50)	24 (19)	**0.008**	**0.010**	0.070	33 (27)	61 (49)	30 (24)	**0.023**	0.920	**0.014**
PR status												
Negative	22 (44)	17 (34)	11 (22)	χ^2^ = 0.93	χ^2^ = 0.02	χ^2^ = 1.06	22 (44)	17 (34)	11 (22)	χ^2^ = 2.52	χ ^=2^ = 0.46	χ^2^ = 2.11
Positive	114 (36)	123 (39)	76 (24)	0.340	0.910	0.300	104 (33)	139 (45)	68 (22)	0.120	0.500	0.150
NPI groups												
Good	21 (15)	16 (12)	12 (14)	χ^2^ = 3.75	χ^2^ = 1.39	χ^2^ = 4.80	21 (43)	17 (35)	11 (22)	χ^2^ = 2.46	χ ^=2^ = 1.36	χ^2^ = 2.40
Moderate	115 (38)	121 (39)	71 (23)	0.163	0.490	0.090	104 (34)	136 (45)	64 (21)	0.117	0.508	0.120
Poor	0 (0.0)	3 (43)	4 (57)				1 (14)	3 (43)	3 (43)			
Oncotype DX score groups												
Low risk	51 (28)	81 (45)	48 (27)	χ^2^ = 11.40	χ^2^ = 0.16	χ^2^ = 12.71	59 (42)	81 (58)	38 (21)	χ^2^ = 0.87	χ^2^ = 0.42	χ^2^ = 0.56
Intermediate risk	63 (46)	44 (32)	29 (21)	**0.002**	0.920	**0.002**	49 (46)	57 (54)	30 (22)	0.349	0.810	0.760
High risk	22 (47)	15 (32)	10 (21)				18 (50)	18 (50)	11 (23)			
*HER2* mRNA clusters												
Low	64 (59)	35 (32)	10 (9)	χ^2^ = 16.50	χ^2^ = 6.45	χ^2^ = 33.10						
High	51 (26)	84 (42)	64 (32)	**<0.001**	**0.014**	**<0.001**	NA	NA	NA	NA	NA	NA

DCIS, ductal carcinoma *in situ*; NST, no special type of breast cancer; LVI, lymphovascular invasion; NPI, Nottingham prognostic index; PR: progesterone receptor; HER2, human epidermal growth factor receptor 2; NA, not available.

* The cohort contained few cases of pleomorphism 1, which have been added to score 2 for statistical analysis purposes to avoid bias. Significant p values are in bold.

^a^ Significant *P*‐values between HER2 immunohistochemistry (IHC) score 0 and score 1+.

^b^ Significant *P*‐values between HER2 IHC score 1+ and HER2 score 2+.

^c^ Significant *P*‐values between HER2 IHC score 0 and both HER2 IHC scores 1+ and 2+ (HER2‐low).

Within the external cohorts used, there was a significant correlation between *HER2* mRNA level and different HER2 IHC scores (from 0 to 3 and HER2‐low only) and *HER2* gene copy number in TCGA and METABRIC cohorts with *P* < 0.001 (Supporting information Figures S5 and S6).

## Discussion

Accurate assessment of HER2 status is integral to the care of patients with BC. Recognising this, the ASCO/CAP HER2 working group released their guideline recommendations on HER2 testing in 2007, which were updated thereafter to provide clearer guidance for HER2 testing and assessment.

At least 16 scenarios of HER2 expression patterns exist when considering the combination of staining intensity (faint, weak, moderate and strong), membrane completeness (complete versus incomplete) and the cut‐off (e.g.10%) used to classify the percentage of HER2 in the invasive tumour cells into two main categories. However, not all the scenarios have been defined (see below) which, in turn, led to a degree of subjectivity and discordance in HER2 scoring. Some studies indicated that the concordance rates among pathologists remains low,^15^, ^28^, ^29^, ^30^ raising concern regarding the need to refine the scoring criteria. Moreover, the distinction between HER2 IHC score 0 from score 1+ was not clinically relevant, and for practical purposes these two groups have often been combined and/or used alternatively in routine practice. Fernandez *et al*. demonstrated that the current standard assays utilised in the clinical setting do not efficiently differentiate IHC scores 0 or 1+ and only 26% of these cases had 90% concordance agreement.^16^ Also, Schettini and colleagues showed that multi‐rater overall kappa score was 0.7, equivalent to substantial agreement, and almost half the discordant cases were between IHC score 0 versus 1+.^31^

All previous attempts for the definition aimed at separating HER2‐positive from HER2‐negative BC for therapeutic and prognostic purposes,^5^, ^6^, ^27^, ^32^, ^33^ as patients with tumours that show low or moderate levels of HER2 protein expression without confirmed gene amplification are currently not candidates for anti‐HER2 agents.^9^ This category, which accounts for 45–55% of BC, is known as HER2‐low class BC, which include IHC score 1+ or 2+ with non‐amplified *HER2* gene by ISH.^34^, ^35^ With the promising response rate of ADC in HER2‐low BC patients,^12^, ^36^, ^37^, ^38^, ^39^ we hypothesised that refining the definition of HER2‐low‐positive class with precise scoring criteria for this group will lead to better scoring concordance levels and better personalisation of ADC therapy.

Borderline HER2‐low BC can be demarcated from HER2‐positive cases through gene amplification assays, but the lower limit of protein expression beyond which the tumour is considered HER2‐negative is not fully identified. In this study, we aimed to refine the definition of different HER2 scoring categories through providing a clearer, easier and applicable interpretation approach for different HER2 expression scenarios. We also sought to provide a definition for HER2‐low‐positive BC through distinguishing HER2 IHC score 1+ from score 0 by using the mRNA expression as ground truth. The rationale behind using mRNA level to dichotomise our cases instead of the patient outcome is that at this low level of protein expression, *HER2* is not the driver oncogene and the clinical behaviour of the tumour and outcome is typically not dependent upon activation of the HER2 pathways.^31^, ^40^ This was supported by Denkert *et al*., who demonstrated that there was no difference between HER2‐low and HER2‐negative tumours in the triple‐negative BC cohort.^41^

Multiple studies show that rates of concordance for HER2 between core biopsy and excision specimens of 98 to 99% are achievable.^42^, ^43^, ^44^ We have demonstrated that *HER2* mRNA was reliable in reflecting HER2 protein level both on core biopsy and full‐face sections. Our results revealed that *HER2* mRNA is statistically significant in differentiating not only HER2‐ positive from ‐negative BC, but also in the HER2‐low class, where it can separate them into two distinct groups and which are correlated with IHC protein level and gene amplification. Our study also showed that HER2 mRNA significantly correlates with HER2 protein and gene amplification levels supported by data from TCGA and METABRIC cohorts. This was supported in other studies that showed high concordance threshold between *HER2* mRNA and IHC and gene amplification.^45^, ^46^, ^47^, ^48^, ^49^ The discrepancy between mRNA level and IHC score that was observed in a few cases could be explained by intratumoral heterogeneity and the ratio of malignant to non‐malignant cells within tumours, which can dilute the influence of the tumour cells on the result, leading to a false‐low mRNA level,^39^, ^50^ while a false‐high mRNA level in HER2 score 0 cases was due mainly to the presence of HER2 expression within the *in‐situ* component.

We have described 10 possibilities for the HER2 expression patterns in BC tumour cells related to the staining intensity, localisation and circumferential staining completeness. Using a trained ANN model, we identified which pattern has the highest weight to differentiate HER2 score 1+ from score 0 based on the ground truth represented by the mRNA level. We found that, at faint intensity, the percentage of expression was more effective than the membranous pattern of expression, whether complete or incomplete. Based on our data, any faint HER2 protein expression in 20% or more can be considered as IHC score 1+. For weak staining, our results were consistent with the 2007 ASCO/CAP HER2 guidelines^27^ and updated UK guidelines^5^ in the definition of HER2 1+ (weak complete staining less than 10% and weak incomplete more than 10%, respectively).

The established algorithm for HER2 scoring according to ASCO/CAP guidelines encompasses 10 of the 16 possible scenarios for HER2 expression patterns. In this study, we tried to complete the missing HER2 expression possibilities based on the current study results, data from the various published HER2 scoring guideline recommendations and our personal experience. Although most of these undefined scenarios are infrequent, such as strong incomplete expression and moderate complete less than 10%, providing more objective criteria and adding more guidance to their scoring would improve the concordance rate among pathologists and consequently better HER2 categorisation and management decision‐making.

To guarantee high interobserver agreement, the magnification rule was also used to define faint staining which comprise areas showing barely visible expression defined as membranous staining confirmed only at ×40, corresponding to faint intensity. This rule is applied and efficient in the assessment of HER2 in gastric carcinoma.^25^

Although H‐score showed a significant association with HER2 scores, we did not include this as a parameter to refine HER2‐low definition. H‐score has been used for assessment of HER2 expression in previous studies, although it is not approved for routine clinical work.^51^, ^52^, ^53^ The limitation of using the H‐score is the non‐linearity of the score, which is due to the heavier weighting of higher‐intensity staining over lower‐intensity staining to calculate the score. One more fallacy of using the H‐score in the assessment of HER2 expression is that it cannot address faint intensity and completeness of membranous staining. Thus, the H‐score, which was designed as a standard scoring scheme to provide continuous scores, is not well suited for the scoring HER2 in BC^51^ and would provide more lack of clarity to pathologists and clinicians.

Based on the refined score, the proportion of HER2 score 1+ cases decreased by 5%, in comparison with the original ASCO/CAP definition that was used in the original scoring in the clinical setting. This could be explained by increasing the cut‐off from the 10% used in clinical practice to 10–20% in the faint category. From this, we can assume that there could be a false increase in score 1+ category in the recent guidelines which may have affected the response rate for ADC in HER2 score 1+ BC patients. The refined score was more efficient in predicting HER2 score 1+ than the current applied score.

The interobserver agreement between HER2 scores based on existing guidelines showed substantial concordance. This magnitude of concordance is in line with others’ reproducibility studies.^16^, ^31^ Schettini and colleagues showed that multi‐rater agreement was substantial, and almost half the discordant cases were between IHC score 0 versus IHC score 1+.^31^ Moreover, in the Phase Ib trastuzumab–deruxtecan study, the concordance between local and central pathology was 70% for HER2 IHC score 1+.^12^

The inter‐ and intra‐observer agreement for the two scoring sessions, according to our refined criteria, was near‐perfect, with reduction of discordant cases between HER2 scores 1+ versus 0 by more than 70%. These results support the fact that current scoring criteria for HER2 scores 1+ and 0 are subjective and less reproducible among pathologists. Guidelines should be updated or refined to distinguish between HER2 scores 0 and 1+, especially in the upcoming era of ADC therapy. Recent studies revealed that 40% of patients with HER2‐low BC achieved partial response to T‐DXD.^12^, ^54^

The refined score showed a stronger association with the clinicopathological parameters than the current applied score. Also, it showed statistical significance with Oncotype DX scores. Our results agreed with both Schettini *et al*. and Tan *et al*., who declared that HER2‐low BC is apparently more associated with axillary lymph node involvement compared to HER2 score 0 tumours.^31^, ^55^ Overall, HER2 protein expression and mRNA level in IHC 1+ category was associated with low tumour grade, low mitotic count, special histological types of BC and low risk of recurrence based on Oncotype DX, as described in other studies.^31^, ^41^, ^55^

This study has some limitations, including that the mRNA levels were measured on full‐face sections, whereas the IHC score was assessed on core biopsy. To overcome this issue, we selected cases with conflicting *HER2* mRNA expression and IHC scores and restained them on resection specimen blocks. The cohort had a low number of outcome events in terms of BC‐related deaths or disease recurrence, so outcome analysis and therapy effects were not feasible in this cohort. Therefore, we have used the mRNA level as our ground truth in classifying patients. Due to the study design, the cohort did not include ER‐negative BC. However, this study aimed at refining the scoring of HER2 protein expression, rather than assessing its oncogenic effect or its interaction with other proteins; thus, we believe that the refined scoring criteria can be generalised and applied to ER‐negative tumours.

## Conclusion

This is the first study to discuss refining the HER2‐low‐positive BC focusing upon the distinction between IHC score 1+ IHC score 0 to provide a more reproducible and non‐arbitrary scoring criteria compared with the current definition, which is more subjective. *HER2* mRNA level is strongly correlated with HER2 protein expression. Further investigations and clinical trials using ADC in HER2‐low‐class BC using the refined criteria is warranted.

## Conflicts of interest

The authors declare no conflicts of interest.

## Supporting information


**Figure S1.** Box blot showing different patterns of HER2 expression in HER2 low category with their median, minimum and maximum values.

**Figure S2.** **A**: Box plot demonstrating distribution of HER2 mRNA among HER2 IHC scores. **B**: Distribution of HER2 IHC scores in the original clinical sore and our score according to refined criteria.

**Figure S3.** Graph showing ANN model prediction of HER2 1+ cut‐off points.

**Figure S4.** Chart illustrating which HER2 score is more accurate in predicting score 1+ based on ROC curve AUC, true positive rate and false positive rate.

**Figure S5.** Graphs showing relation between ERBB2 RNA level, HER2 protein level in the form of IHC expression and HER2 gene copy number. **A**: ERBB2 RNA level significantly correlates with HER2 IHC scores (0‐3+) and HER2 low cases**, B.** Positive linear correlation between ERBB2 RNA level and HER2 gene copy number in all HER2 scores and in HER2 low cases as shown in **C** and **D**, respectively.

**Figure S6.** Box plot chart showing significant the correlation between HER2 RNA level and HER2 IHC score in TCGA cohort. **A**: ALL HER2 IHC scores from (0‐3+), while **B** shows HER2 low cases only

**Table S1.** Clinicopathologic characteristics of the study cohort.
**Table S2.** Description of HER2 expression patterns.
**Table S3**. Associations between clinicopathologic parameters and HER2 mRNA clusters.

## Data Availability

All data used in this study are available and can be accessed upon reasonable request. The following publicly available data sets were used at: https://identifiers.org/cbioportal:brca_tcga; https://identifiers.org/cbioportal:brca_metabric
