# Supplementary material for: Refining the definition of HER2‐low class in invasive breast cancer
Source: Histopathology. 2022 Sep 12;81(6):770–85. doi: 10.1111/his.14780 (PMC9826019; doi:10.1111/his.14780)
Supplement: Supplementary file 1 — Figure S1. Box blot showing different patterns of HER2 expression in HER2 low category with their median, minimum and maximum values. [file HIS-81-770-s005.docx]

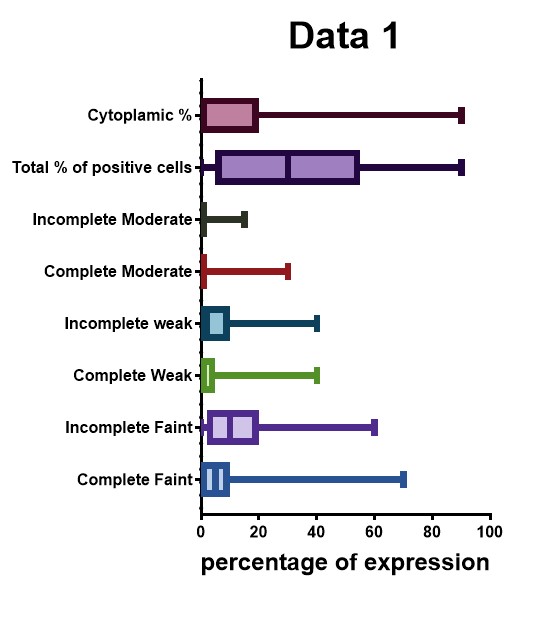


**Supplementary Figure 1**: Box blot showing different patterns of HER2 expression in HER2 low category with their median, minimum and maximum values.
