# Supplementary material for: Refining the definition of HER2‐low class in invasive breast cancer
Source: Histopathology. 2022 Sep 12;81(6):770–85. doi: 10.1111/his.14780 (PMC9826019; doi:10.1111/his.14780)
Supplement: Supplementary file 2 — Figure S2. A: Box plot demonstrating distribution of HER2 mRNA among HER2 IHC scores. B: Distribution of HER2 IHC scores in the original clinical sore and our score according to refined criteria. [file HIS-81-770-s001.docx]

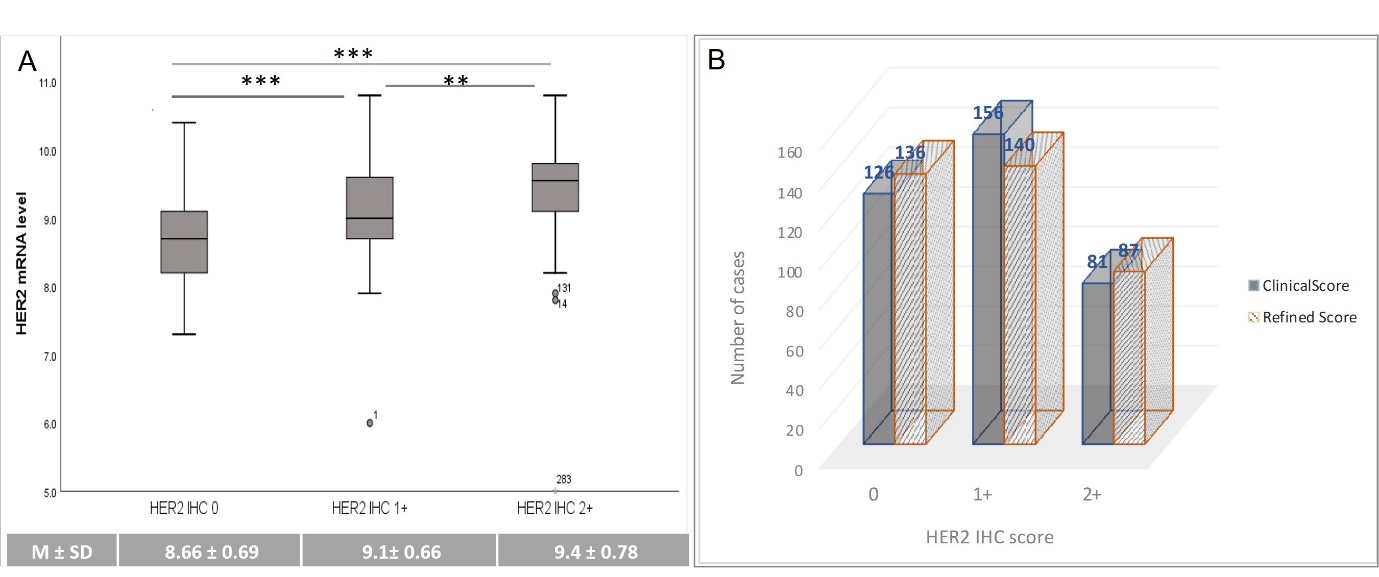


**Supplementary Figure 2: A**: Box plot demonstrating distribution of HER2 mRNA among HER2 IHC scores. **B**: Distribution of HER2 IHC scores in the original clinical sore and our score according to refined criteria.
