# Supplementary figures and images for: Refining the definition of HER2‐low class in invasive breast cancer
Source: Histopathology. 2022 Sep 12;81(6):770–85. doi: 10.1111/his.14780 (PMC9826019; doi:10.1111/his.14780)

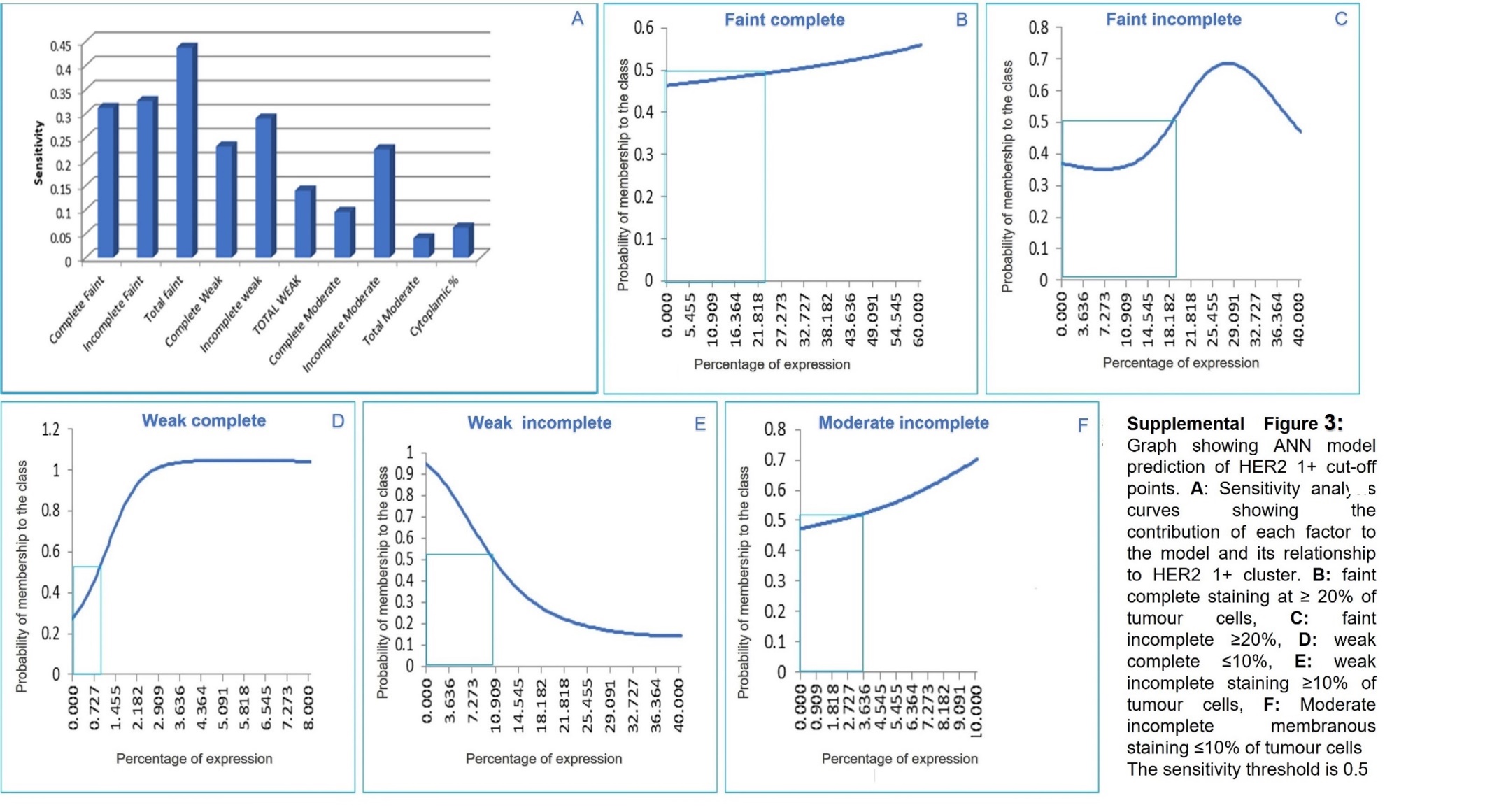

Supplement: Supplementary file 3 — Figure S3. Graph showing ANN model prediction of HER2 1+ cut‐off points. [file HIS-81-770-s003.docx]
