# Supplementary material for: Refining the definition of HER2‐low class in invasive breast cancer
Source: Histopathology. 2022 Sep 12;81(6):770–85. doi: 10.1111/his.14780 (PMC9826019; doi:10.1111/his.14780)
Supplement: Supplementary file 4 — Figure S4. Chart illustrating which HER2 score is more accurate in predicting score 1+ based on ROC curve AUC, true positive rate and false positive rate. [file HIS-81-770-s007.docx]

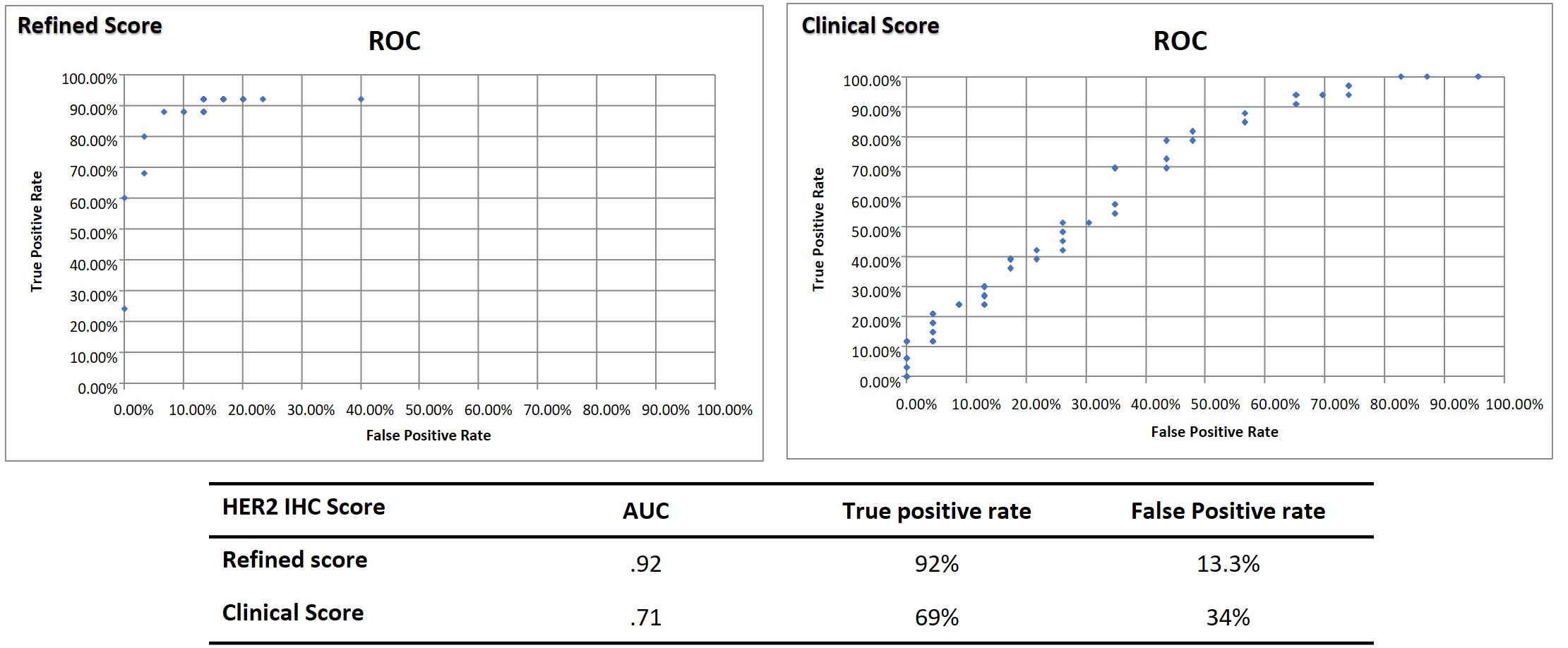


**Supplementary figure 4:** Chart illustrating which HER2 score is more accurate in predicting score 1+ based on ROC curve AUC, true positive rate and false positive rate.
