# Supplementary material for: Refining the definition of HER2‐low class in invasive breast cancer
Source: Histopathology. 2022 Sep 12;81(6):770–85. doi: 10.1111/his.14780 (PMC9826019; doi:10.1111/his.14780)
Supplement: Supplementary file 5 — Figure S5. Graphs showing relation between ERBB2 RNA level, HER2 protein level in the form of IHC expression and HER2 gene copy number. A: ERBB2 RNA level significantly correlates with HER2 IHC scores (0‐3+) and HER2 low cases, B. Positive linear correlation between ERBB2 RNA level and HER2 gene copy number in all HER2 scores and in HER2 low cases as shown in C and D, respectively. [file HIS-81-770-s004.docx]

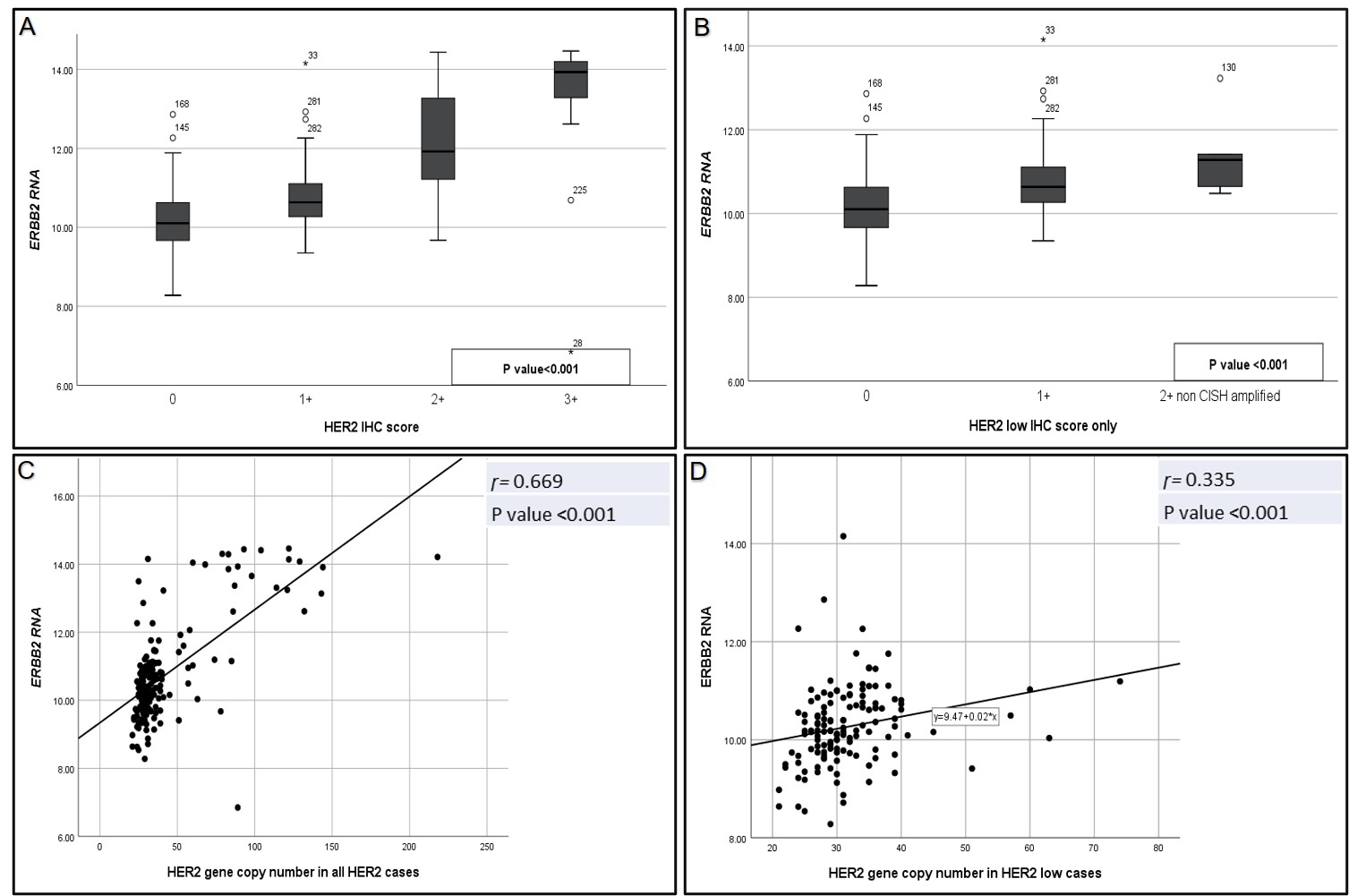


**Supplementary Figure 5**: Graphs showing relation between ERBB2 RNA level, HER2 protein level in the form of IHC expression and HER2 gene copy number. **A**: ERBB2 RNA level significantly correlates with HER2 IHC scores (0-3+) and HER2 low cases**, B.** Positive linear correlation between ERBB2 RNA level and HER2 gene copy number in all HER2 scores and in HER2 low cases as shown in **C** and **D**, respectively.
