# Supplementary material for: Refining the definition of HER2‐low class in invasive breast cancer
Source: Histopathology. 2022 Sep 12;81(6):770–85. doi: 10.1111/his.14780 (PMC9826019; doi:10.1111/his.14780)
Supplement: Supplementary file 6 — Figure S6. Box plot chart showing significant the correlation between HER2 RNA level and HER2 IHC score in TCGA cohort. A: ALL HER2 IHC scores from (0‐3+), while B shows HER2 low cases only [file HIS-81-770-s002.docx]

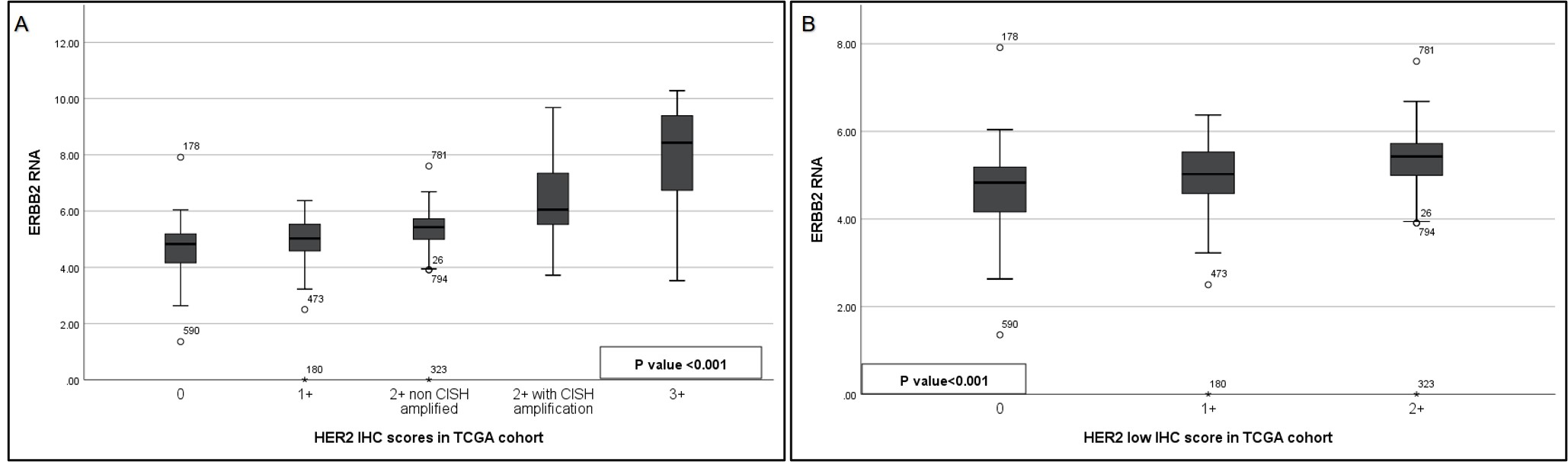


**Supplementary Figure 6**: Box plot chart showing significant the correlation between HER2 RNA level and HER2 IHC score in TCGA cohort. **A**: ALL HER2 IHC scores from (0-3+), while **B** shows HER2 low cases only
