# Supplementary material for: Refining the definition of HER2‐low class in invasive breast cancer
Source: Histopathology. 2022 Sep 12;81(6):770–85. doi: 10.1111/his.14780 (PMC9826019; doi:10.1111/his.14780)
Supplement: Supplementary file 7 — Table S1. Clinicopathologic characteristics of the study cohort. Table S2. Description of HER2 expression patterns. Table S3. Associations between clinicopathologic parameters and HER2 mRNA clusters. [file HIS-81-770-s006.docx]

**Supplementary Table 1:** Clinicopathologic characteristics of the study cohort

| Parameter | Number of cases (%) |
| --- | --- |
| Age at diagnosis (years)  < 50  ≥ 50 | 82 (23)  281 (77) |
| Menopausal status  Pre  post | 101 (28)  262 (72) |
| Tumour size (cm)  < 2.0  ≥ 2.0 | 167 (46)  196 (54) |
| Associated DCIS  No  Yes | 110 (30)  253 (70) |
| Histologic tumour type  Invasive Carcinoma of no special type  Other types | 233 (64)  130 (36) |
| Histologic tumour types  NST  Lobular  Tabular  Special type  Metaplastic carcinoma | 233 (64)  55 (15)  11 (3)  58 (16)  6 (2) |
| Tumour grade  1  2  3 | 23 (6)  203 (56)  137 (38) |
| Lympho-vascular Invasion  No  Yes | 288 (79)  75 (21) |
| Lymph node status*  Negative  Positive | 237 (65)  125 (35) |
| PR status  Negative  Positive | 50 (14)  313 (86) |
| Nottingham prognostic index*  Good prognostic group  Moderate prognostic group  Poor prognostic group | 49 (14)  306 (84)  7 (2) |
| Oncotype DX score group  Low risk <18  Intermediate risk (≥18 <31)  High risk ≥31 | 180 (50(  136 (38)  47 (13) |

***** Total number of cases 362/363

**Supplementary Table 2:** Description of HER2 expression patterns

| HER2 expression patterns | Total Cases (n=363) | |
| --- | --- | --- |
|  | **Frequency**  **N (%)** | **Median/Range** |
| Complete Faint | 209 (58) | 5 (0-70) |
| Incomplete Faint | 279 (78) | 10 (0-60) |
| Complete Weak | 177 (49) | 0 (0-40) |
| Incomplete weak | 184 (51) | 2 (0-40) |
| Complete Moderate | 28 (8) | 0 (0-30) |
| Incomplete Moderate | 16 (4) | 0 (0-15) |
| Total percentage of positive cells | 294 (81) | 30 (0-90) |
| Cytoplasmic expression | 135 (37) | 0 (0-90) |
| H score | 294 (81) | 16 (0.5-105) |

**Supplementary table 3: Associations between clinicopathologic parameters and HER2 mRNA clusters**

| Parameter | HER2 negative cluster | HER2 positive cluster | P Value* |
| --- | --- | --- | --- |
|  | **Score 0**  **(N, %)** | **Score 1**  **(N, %)** |  |
| Age at diagnosis (yrs)  <50  ≥ 50 | 32 (44)  77 (33) | 40 (56)  159 (67) | X^2^=3.36  0.066 |
| Menopausal status  Pree  post | 37 (42)  72 (33) | 51 (58)  148 (67) | X^2=^2.39  0.12 |
| Tumour size (cm)  <2  ≥ 2 | 55 (39)  54 (32) | 87 (61)  112 (68) | X^2^=1.28  0.256 |
| Associated DCIS  No  Yes | 19 (20)  90 (42) | 74 (80)  125 (58) | **X^2=^13.04**  **<0.001** |
| Histologic tumour type  NST  Other types | 84 (43)  25 (23) | 113 (57)  86 (77) | **X^2^=12.56**  **<0.001** |
| Histologic tumour types  NST  Lobular  Tabular  Special type  Metaplastic carcinoma | 84 (43)  10 (20)  1 (11)  13 (27)  1 (20) | 113 (57)  39 (80)  8 (89)  35 (73)  4 (80) | **X^2=^7.36**  **0.007** |
| Histological tumour grade  1  2  3 | 3 (15)  42 (25)  64 (53) | 17 (85)  126 (75)  56 (47) | **X^2^=28.46**  **<0.001** |
| Tubule Formation  1  2  3 | 1 (9)  16 (31)  92 (38) | 10 (91)  36 (69)  153 (62) | X^2^=4.31  0.116 |
| Pleomorphism  2  3 | 28 (24)  81 (43) | 91 (76)  108 (57) | **X^2^=11.93**  **0.001** |
| Mitosis  1  2  3 | 36 (21)  41 (51)  32 (55) | 134 (79)  39 (49)  26 (45) | **X^2^=33.72**  **<0.001** |
| LVI  No  Yes | 85 (34)  24 (41) | 164 (66)  35 (59) | X^2^=0.89  0.345 |
| Lymph node Status  Negative  Positive | 81 (39)  27 (27) | 125 (61)  74 (73) | **X^2^= 4.70**  **0.030** |
| PR status  Negative  Positive | 18 (39)  91 (35) | 28 (61)  171 (65) | X^2^=0.33  0.57 |
| NPI groups  Good  Moderate  Poor | 11 (26)  95 (37)  2 (29) | 31 (74)  163 (63)  5 (71) | X^2^=1.92  0.381 |
| Oncotype DX score Groups  Low risk  Intermediate risk  High risk | NA | NA | NA |
| *HER2* mRNA clusters  Low  High | NA | NA | NA |

**DCIS:** Ductal carcinoma in situ**; NST:** No special type breast cancer**, LVI:** lymphvascular invasion, **NPI:** Nottingham Prognostic index; **PR**: Progesterone receptor
